# Smoking among adolescents is associated with their own characteristics and with parental smoking: cross-sectional study

**DOI:** 10.1590/1516-3180.2017.0154220717

**Published:** 2017-11-17

**Authors:** Rafaela Campos Cuissi de Andrade, Aline Duarte Ferreira, Dionei Ramos, Ercy Mara Cipulo Ramos, Catarina Covolo Scarabottolo, Bruna Thamyres Ciccotti Saraiva, Luis Alberto Gobbo, Diego Giulliano Destro Christofaro

**Affiliations:** I MSc. Physiotherapist, Department of Physiotherapy, School of Technology and Sciences, Universidade Estadual Paulista Julio de Mesquita Filho (UNESP), Presidente Prudente (SP), Brazil.; II MSc. Professor, Department of Physical Education, Universidade do Oeste Paulista (UNOESTE), Presidente Prudente (SP), Brazil.; III Professor, Department of Physiotherapy, Universidade Estadual Paulista Julio de Mesquita Filho (UNESP), School of Technology and Sciences, Presidente Prudente (SP), Brazil.; IV Master’s Student, Department of Physical Education, Universidade Estadual Paulista Julio de Mesquita Filho (UNESP), Instituto de Biociências Campus de Rio Claro, Rio Claro (SP), Brazil.; V Professor, Department of Physical Education, School of Technology and Sciences, Universidade Estadual Paulista Paulista Julio de Mesquita Filho (UNESP), Presidente Prudente (SP), Brazil.

**Keywords:** Adolescent, Smoking, Parents

## Abstract

**BACKGROUND:**

This study aimed to analyze the association between smoking during adolescence and the characteristics of smoking and alcohol consumption among their parents.

**DESIGN AND SETTING:**

Cross-sectional study in Londrina (PR), Brazil.

**METHODS:**

The subjects comprised 1,231 adolescents aged 14-17 years. The adolescents and their parents answered a self-report questionnaire that asked for sociodemographic information and data on smoking and alcohol consumption. Multiple logistic regression models were used to analyze associations between smoking among adolescents and their characteristics (age, sex, period of the day for attending school, alcohol consumption and socioeconomic level) and their parents’ characteristics (smoking, alcohol consumption, age and education level), adjusted according to the adolescents’ characteristics (sex, age and socioeconomic level).

**RESULTS:**

The prevalence of smoking among adolescents was 3.4% (95% confidence interval, CI: 2.4-4.4). Adolescents whose mothers or fathers were smokers were 2.0 and 2.5 times more likely to be smokers, respectively. The prevalence of smoking among adolescents with a smoking mother was 7.1% (95% CI: 2.6-10.7) and a smoking father, 5.4% (95% CI: 1.6-8.5). There were significant associations between smoking adolescents and age [5.2% (95% CI: 3.3-6.6)], studying at night [9.6% (95% CI: 4.0-15.5)] and alcohol consumption [69.0% (95% CI: 55.0-83.0)]. It was observed that the number of alcoholic beverage doses consumed was higher among smoking adolescents (P = 0.001).

**CONCLUSION:**

Adolescent smoking was associated with smoking by their parents, regardless of the gender of the parents or adolescents. Age, alcohol consumption and studying at night are characteristics of adolescents that can contribute towards smoking.

## INTRODUCTION

Smoking is considered to be a behavior that puts health at risk.^1^ Several studies have demonstrated a strong relationship between smoking and various types of diseases in the adult population, such as carotid calcification^2^ and other cardiovascular problems, like stroke.^3^ However, this type of behavior has been detected not only among adults, but also in young populations. In a study conducted in Saudi Arabia, Al-Zalabani and Kasim^4^ observed that the prevalence of smoking was around 15% among the young people who they evaluated.

Adolescent smoking is increasing in poorer countries. Smoking in adulthood may start during adolescence, which demonstrates the importance of studies addressing this issue.^5^ Tavares et al.^6^ and Barreto et al.^7^ highlighted that adolescence is a period of great exposure and vulnerability to consumption of substances such as tobacco and alcohol, with frequent experimentation by adolescents. Consequently, determining the factors that could cause this type of behavior among adolescents is important. Another aspect that has been investigated is whether adolescents’ household environment might contribute to such behavior.

In a study on adolescents aged 13-18 years, Vázquez-Rodríguez et al.^8^ reported that parental smoking was associated with smoking among their children. Similar results were observed in some studies in which adolescent smoking was more prevalent among those whose parents were smokers.^9^^,^^10^ Tondowski et al.^9^ showed that approximately 45% of adolescents who reported frequent tobacco use had fathers or mothers who smoked. In addition, it has been shown that smoking during adolescence may be linked both to use of illicit drugs such as marijuana and to use of licit drugs such as alcohol.^11^^,^^12^^,^^13^^,^^14^^,^^15^

The majority of previous studies have only examined parental smoking as a risk factor. However, other lifestyle variables such as alcohol consumption and sociodemographic characteristics such as the parents’ ages and educational levels, also need to be considered. One of the hypotheses is that the parents’ characteristics other than smoking may also be associated with smoking among adolescents. Moreover, it needs to be emphasized that the characteristics of the adolescents themselves should also be considered in order to eliminate possible confounding factors, since late adolescence^16^ and being male^17^ tend to be more associated with smoking, and socioeconomic level may also be associated, depending on the characteristics of each country.^18^

Studies that investigate lifestyle habits between parents and children can contribute towards health promotion actions, if these relationships are observed in the family environment. Therefore, the aim of the present study was to analyze the association between smoking during adolescence and the lifestyle characteristics (smoking and alcohol consumption) of parents or family members who live with adolescents.

## METHODS

### Sample

The sample of this study formed part of a larger study that looked at risk factors for health among adolescents at public schools in the city of Londrina (PR), Brazil, and among their parents. To contact the adolescents, the Londrina Department of Education was first contacted in order to explain the objectives of the study. Subsequently, the Department identified the six largest public schools in the central region, which receive adolescents from different areas of the city (north, south, east, west and central areas). Subsequently, the researchers contacted the principals of the schools that were invited to participate in the study to explain the objectives of the study. After authorization from the schools’ directors, contact was made with all classes of students aged 14-17 years in these schools.

To calculate the sample, the prevalence of smoking among adolescents was taken to be 15%,^4^ and a tolerable error of 3% and power of 80% were used. Since the sample was selected through clusters, a design correction of 1.5 was used. To anticipate possible losses from the sample, 10% was added to the sample calculation. Thus, the minimum sample required was 870 adolescents. In the end, the study included 1231 adolescents (716 girls and 515 boys) aged 14-17 years.

Adolescents and their parents or family members who agreed to participate in the study signed a free and informed written consent form. This study was approved by the Research Ethics Committee of the institution responsible for this study (procedural number: 0.181.0.268.000-10; register number: 367.801).

The adolescents took the consent form home for their parents to sign and thus authorize the adolescent to participate in the study. Along with this consent form, they also took the parents’ questionnaire with them, so that their parents could answer this instrument at home. The parents’ questionnaire contained questions about their lifestyle habits (among them smoking and alcohol consumption) and sociodemographic variables (sex, age and schooling level). In total, 1,202 mothers and 871 fathers answered the questionnaire. Subsequently, the adolescents were evaluated at school.

### Smoking and alcohol

Smoking status was ascertained through analysis of participants’ smoking behavior.^19^ If individuals replied that they had smoked cigarettes within the previous 30 days, they were considered to be smokers. The number of cigarettes that these individuals consumed in a typical week was also established.

Alcohol consumption was obtained through questions based on the questionnaire of the Brazilian Center for Psychotropic Drugs (CEBRID),^20^ which assesses the frequency and quantity of alcoholic drinks consumed. Adolescents and parents or family members who reported consumption of more than 1-2 doses (each dose corresponded to 250 ml of beer or 40 ml of distilled beverages in this study) on more than 1-2 days a week were classified as high consumers. The cutoff points used in this study were adapted from Moreira et al.^21^ These instruments demonstrate good reproducibility values: kappa = 0.81 for smoking and kappa = 0.83 for alcohol consumption.

### Anthropometric variables

The adolescents were measured wearing light clothing and no shoes. Weight was measured using a portable scale (Plenna; precision of 0.100 kg) with a capacity of 150 kg. Height was measured using a portable stadiometer (Sanny; precision of 0.1 cm) with a scale in centimeters. The anthropometric characteristics of the adolescents were evaluated by two previously trained evaluators. The procedures were applied in accordance with the recommendations of Gordon et al.^22^ To assess the participants’ nutritional status, the body mass index (BMI) was calculated as the ratio between the weight and height squared. The adolescents’ nutritional status was classified in accordance with the values proposed by Cole et al.^23^ Overweight among the parents was determined based on the cutoff points of the World Health Organization, and adults with BMI greater than or equal to 25 kg/m^2^ were classified as overweight.^24^

### Sociodemographic variables

The parents’ educational level was evaluated as the number of years of study reported over the course of their lives. Parental schooling was divided into terciles, such that lower education level was considered to be up to 8 years of study; medium education level, from 8 to 12 years; and higher education level, more than 12 years.

Parental age was determined as the difference between the date of data collection and birth date. Subsequently, age was divided into terciles.

To define the families’ economic class, the 2011 Brazilian economic classification criteria of the Brazilian Market Research Association (ABEP) were used.^25^ Householders’ education level and the presence and quantity of certain rooms, assets and domestic employees in the homes analyzed were considered (color TV, VCR or DVD player, radio, number of bathrooms, car, washing machine, housemaids, refrigerator and freezer). At the end of this instrument, a scoring system is provided in which the individual is classified according to economic strata, such that higher scores represent higher economic strata.

### Statistical analysis

The data characterizing the sample were presented as means and standard deviations stratified according to smoking status (smoker or nonsmoker). Analysis on the association between the dependent variable (smoking adolescents) and the independent variables was performed using the chi-square test.

Subsequently, two multivariate models were created and were analyzed through binary logistic regression. The association between smoking adolescents and their own characteristics (sex, age, period of the day for attending school, day or night, socioeconomic status and alcohol consumption) was analyzed. The association between smoking adolescents and the characteristics of their mothers and fathers was analyzed. In the first model, unadjusted smoking among adolescents was analyzed in relation to their mothers and fathers’ smoking, alcohol consumption, age and education level. In the second model, which was adjusted according to the adolescents’ characteristics, the sociodemographic variables of the adolescents that might be potential confounders were considered (sex, age and socioeconomic status). Although only the adolescents’ ages presented P values lower than 0.200, it was decided that, in analyzing the association with smoking, the adolescents’ sex and socioeconomic status would be inserted as adjustment variables. This was done to ascertain whether the possible associations between smoking and the variables analyzed would be independent of these confounding factors.

The significance level used for all analyses was P ≤ 5%. The confidence interval (CI) used was 95%. The analyses were performed using the Statistical Package for the Social Sciences (SPSS) software, version 15.0.

## RESULTS

The prevalence of smoking in the sample of this study was 3.4% (95% CI: 2.4-4.4), which was equivalent to 42 adolescents. The average number of cigarettes smoked by the adolescents interviewed was 0.29 per month with no difference between boys and girls (P = 0.351). The prevalence of smoking among the mothers was 12.1% (95% CI: 10.5-14.2) and among the fathers, 17.9% (95% CI: 15.3-20.4). Smoking mothers consumed more alcohol than did mothers who did not smoke. Fathers who smoked presented lower weight, lower BMI and higher alcohol consumption than did fathers who did not smoke. Mothers and fathers with fewer years of schooling were more likely to be smokers. The prevalence of smoking was higher among fathers and mothers of medium socioeconomic status. Table 1 presents information regarding sample characterization. It can be seen that the highest average of alcoholic beverages consumed in doses were higher among smoking adolescents.

**Table 1.**
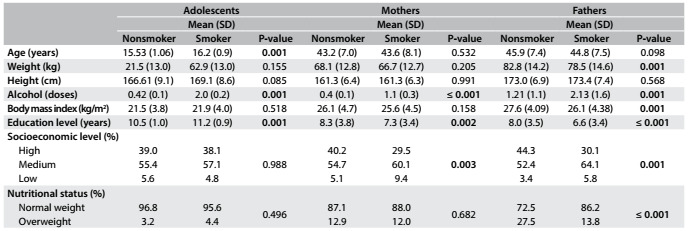
Characteristics of the sample according to smoking status

SD = standard deviation.

Table 2 shows the significant associations between smoking among adolescents and later adolescence, studying at night and alcohol consumption. Older adolescents (16-17 years) presented higher levels of smoking behavior (5.2%; 95% CI: 3.45-6.82) than younger adolescents (1.6%; 95% CI: 0.56-2.61) (P = 0.002). The adolescents who studied in the evenings presented higher prevalence of smoking (9.6%; 95% CI: 4.0-15.5) than those who studied during the day (3.2%; 95% CI: 1.94-3.91). Smoking adolescents presented higher frequency of alcohol consumption: among the 42 adolescents who smoked, 29 (69.0%; 95% CI: 55.0-83.0) consumed alcohol and 7.0% of them (95% CI: 0.65-14.95) consumed alcohol with a frequency of four times a week.

**Table 2.**
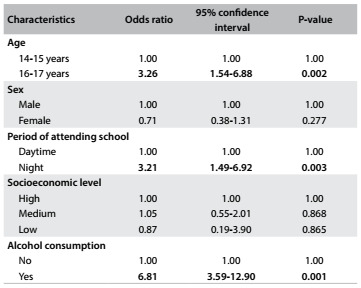
Association between adolescents’ smoking habits and their characteristics

Table 3 shows the associations between adolescents who smoked and mothers or female guardians who smoked. Adolescents whose mothers were smokers were twice as likely to have this habit. The prevalence of smoking among adolescents with smoking mothers was 7.1% (95% CI: 2.6-10.7), compared with 2.3% (95% CI: 1.85-3.86) among adolescents with non-smoking mothers. In both the raw and adjusted analyses on the variables relating to the adolescents, associations between smoking adolescents and alcohol consumption could be seen. There were no significant differences in smoking levels among adolescents between those with older mothers and those with younger mothers, or between those with mothers with lower education levels and those with mothers with higher education levels.

**Table 3.**
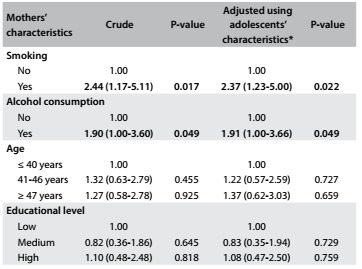
Association between smoking among adolescents and characteristics of their mothers

*Adjusted according to the adolescents’ sex, socioeconomic level and age.

The prevalence of smoking among adolescents with smoking fathers was 5.4% (95% CI: 1.6-8.5). Smoking among fathers was also associated with smoking among adolescents: teens whose fathers smoked were 2.5 times more likely to be smokers (Table 4).

**Table 4.**
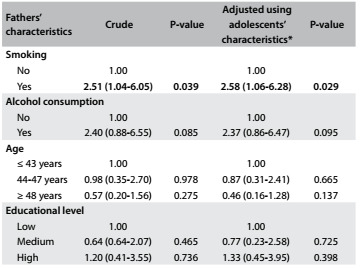
Association between smoking among adolescents and characteristics of their fathers

*Adjusted according to the adolescents’ sex, socioeconomic level and age.

Table 5 presents information on the relationship between smoking among adolescents and the smoking habits of both of their parents. There were no associations between adolescent smoking and both parents smoking.

**Table 5.**
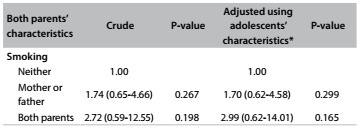
Association between smoking among adolescents and smoking among parents

*Adjusted according to the adolescents’ sex, socioeconomic level and age.

## DISCUSSION

The prevalence of smoking adolescents in this study can be considered low (3.4%) in comparison with other studies.^4^^,^^26^ In a recent study, Figueiredo et al.^16^ observed that the prevalence of smoking in a sample of adolescents aged from 12 to 17 years was 5.7%, considering several Brazilian cities. Their findings were similar to those of the present study and their prevalence can also be considered low. One reason for this low prevalence appears to be related to restrictions on tobacco advertising on the television and to laws prohibiting tobacco use in public places, along with increased prices for cigarettes and increased activity of smoking cessation programs, as shown by Levy et al.^27^

There were no significant differences in the nutritional status of adolescent smokers and nonsmokers in this study. This same relationship was observed for the mothers, but smoking fathers presented lower weight and prevalence of overweight than did nonsmoking fathers. Adolescents are at an early stage of life, at which they have probably not yet established a pattern for nutritional status or smoking habits. Considering the difference in the nutritional status among their fathers, one of the reasons for this that can be considered is nicotine levels, which cause several changes to appetite and metabolic rate, thus giving rise to differences between smokers and nonsmokers.^28^ For both fathers and mothers, those with higher average schooling levels presented lower tobacco consumption than did parents with lower schooling levels, possibly because parents with higher education levels have more knowledge about the harm that cigarette smoking can cause.

This study found that adolescents whose mothers or fathers smoked were about 2.0 and 2.5 times as likely, respectively, to have the same kind of behavior, even after various adjustments for potential confounders. It was observed that the habit of smoking among parents was associated with their children’s habits, independent of the abovementioned variables. In a study on young Canadians, O’Loughlin et al.^29^ also observed that parental smoking was associated with the onset of smoking during their children’s adolescence, regardless of parental schooling levels. Similar relationships have been observed in other studies.^9^^,^^10^

The fact is that teens tend to replicate their parents’ habits. In a recent meta-analysis, Laird et al.^30^ observed that adolescents with physically active parents were more likely to be physically active. However, this replication of habits does not seem to occur for healthy habits alone, and a similar relationship regarding smoking habits is observed between adolescents and their parents. One of the hypotheses for this is that these young people may have felt more freedom to experiment with smoking because of the example seen in their homes.

Having a father and/or mother who smokes seems to represent a permissive attitude for adolescents, based on their parents’ behavior, thus producing an image that smoking is acceptable and possibly contributing towards a process of initiation of smoking. Nonetheless, in our sample, contrary to expectations, there was no association between smoking among adolescents and both parents being smokers. One of the possible reasons for this finding is that the prevalence of occurrences of both parents smoking at home was low: only 4.4%.

An association was observed between mothers who had the behavior of consuming alcohol and smoking among adolescents. One of the possible reasons for this is the strong relationship between smoking and alcohol. Elicker et al. showed that 39.2% of the adolescents in Porto Velho (RO), Brazil, consumed alcohol for the first time at home.^31^ This may be one of the reasons, since alcohol consumption at parties or family meetings would not be characterized as a risk factor for health, but as a normal attitude that could be related to smoking. Perhaps parents who are permissive regarding alcohol use may also be permissive regarding to smoking among young people.

Among the characteristics of adolescent smoking, there were associations with age (being older), alcohol consumption and the period of the day in which adolescents attended school (night). Khuder et al.^32^ found that older adolescents were about six times more likely to be smokers than younger adolescents. One reason for this relationship is the transformation that adolescents experience during this stage of life. This is a period in which social relationships are important, and this could contribute towards starting some types of behavior such as smoking, with the aim of achieving acceptance in social groups. Several studies have reported the strong influence that friends have on smoking habits among adolescents.^10^^,^^33^

Regarding alcohol consumption, those who consumed alcoholic beverages were six times more likely to be smokers. Several studies have demonstrated significant relationships between smoking and alcohol consumption among adolescents.^13^^,^^14^^,^^15^ Among the substances found in large quantities in cigarettes, nicotine acts in many areas of the brain. It has been hypothesized that neuronal nicotinic acetylcholine receptors act in a specific brain area that also causes higher propensity towards alcohol use.^34^

Additionally, adolescents who went to school in the evenings were about three times more likely to have a smoking habit than their peers who attended school during the day. This corroborated the findings of Farias Junior et al.^35^ who observed that young people who attended classes in the evenings presented a greater chance of being smokers. This relationship was also observed among almost 3,000 adolescents in northern Brazil.^36^ Among the reasons that could explain this relationship, the first is that adolescents who study in the evenings have a higher average age than the adolescents who study during the day.

Another characteristic of the adolescents attending school in the evenings is that they tend to work during the day, which aids independence and brings the possibility of buying cigarettes with their own income. The association between adolescents working and smoking has also been reported in another study.^37^ A further factor to be considered as a hypothesis, but which was not analyzed in this study, is that at night, several bars and nightclubs, which are often close to where schools are located, are open. This may contribute towards this type of behavior among young people who attend these places.

The practical application for the present study is that it serves as a reminder to different healthcare agencies regarding the importance of organizing prevention strategies among families, in order to avoid problems caused by smoking in the future. In this regard, the recent findings of West et al.^38^ are noteworthy. Through a cohort study conducted over a period of more than twenty years, these authors found that children exposed to parental smoking had higher odds of developing carotid atherosclerotic plaque in adulthood.

The limitations of the present study were, firstly, that the outcome was assessed using a questionnaire, which was self-administered and may have underestimated the prevalence of smoking, since some adolescents may have omitted the fact that they were smokers. In addition, this was an epidemiological cross-sectional study and it was not possible to quantify serum nicotine levels to confirm the presence of the smoking habit among these adolescents and thus to preclude the limitation of potential underreporting of this habit. Another factor to be mentioned is that the sample was not representative of all schools in the city in which the study was conducted. However, the sample was selected from the six largest schools in the central region of the city of Londrina, and these schools receive students from different areas of the city, with large numbers of students, which made the sample more representative.

The strong aspects of this study are its large sample size, and all of the adjustments made in the analysis on the data. It is worth noting that through stratification of the parents according to sex, it became possible to observe the relationships between both the fathers’ and the mothers’ smoking habits and those of the adolescents.

## CONCLUSION

The smoking habit among adolescents was associated both with parental and maternal smoking, regardless of the gender of the parents or adolescents. Factors such as age, alcohol consumption and attending school at night were characteristics among these adolescents that may have contributed towards smoking. Health promotion actions need to focus on the family unit and not on strategies that are isolated from each other.
